# Ipsilateral breast metastasis after axillary dissection caused by epithelioid sarcoma: a case report and pathological investigation

**DOI:** 10.1186/s13000-019-0888-z

**Published:** 2019-10-15

**Authors:** Luyan Chen, Li Wang, Xiaochen Zhang, Minya Yao, Peifen Fu

**Affiliations:** 1 Department of Breast Center, First Affiliated Hospital, School of Medicine, Zhejiang University, 79 Qingchun Road, Hangzhou, 310009 China; 2 Department of Pathology, Shaoxing People’s Hospital, School of Medicine, Zhejiang University, Shaoxing, 312000 China; 3 Department of Oncology, First Affiliated Hospital, School of Medicine, Zhejiang University, Hangzhou, 310009 China

**Keywords:** Epithelioid sarcoma, Axillary mass, Breast metastasis

## Abstract

**Background:**

Epithelioid sarcoma (ES) is a rare malignant soft tissue tumor, commonly occurring in distal extremities, such as fingers, hands and wrists. For oncologists and surgeons, a female patient with enlarged axillary lymph node on one side only is easily diagnosed with an occult breast carcinoma rather than ES. Besides, whole breast metastasis of ES has not been reported yet.

**Case presentation:**

A 47-year-old Chinese woman came to the outpatient clinic of First Affiliated Hospital of Zhejiang University (FAHZU) with a complaint of an asymptomatic right axillary mass for 3 months. Then she received surgical resection of the right axillary lymph nodes and right supraclavicular lymph nodes. According to the clinical tumor site and routine immunohistochemistry (IHC), suspicion of metastatic epithelial sarcoma and metastatic breast cancer could not be ruled out. Subsequently, with more detailed medical history review and physical examination, a mass on the right forearm was found, which was considered as the primary lesion. Further IHC and Molecular Genetics revealed that all the neoplastic cells exhibited loss of INI1 protein and were negative for ERG gene rearrangement yet positive for epithelial membrane antigen (EMA), cytokeratin (CK) 8, CK19, Vimentin, CD34. The final diagnosis was ES. She received postoperative chemotherapy, without radiotherapy. Unexpectedly, an ipsilateral breast metastasis was developed at ten months after surgery. Regrettably, there was no positive result of the metastatic breast sample, based on a genome sequencing by a 381-cancer-gene panel in a lab. Therefore, she went through another round of chemotherapy and took Apatinib for maintenance treatment. During the last follow-up (26 months after diagnosis), the disease was under control.

**Conclusion:**

This rare but interesting case enables breast surgeons and pathologists to accumulate more experience of differential diagnosis of axillary mass for personalized treatment in clinical practice. Meanwhile, ipsilateral breast metastasis of ES we reported in the case urges that clinicians attach greater importance to the tumor metastasis mechanism.

## Background

Epithelioid sarcoma (ES) is a rare malignant soft tissue tumor originating from mesenchymal tissue with epithelium-like features, which commonly occurs in distal extremities, such as fingers, hands and wrists. It was first described by Enzinger in 1970 [1]. ES grows slowly and presents great metastatic tendency especially in adolescents and young adults. According to case reports, there have been unusual sites with ES metastases such as the vulva [2], scapular region and pelvis [3]. The majority of patients had initial complaints of skin ulcer and mass. Here we reported a rare case of epithelioid sarcoma with an initial symptom of an axillary mass only, which originated from the right forearm and developed a breast metastasis after a ten-month progression. This case provides information and experience about the diagnosis and treatment of axillary mass for oncologists, surgeons and pathologists, as well as a deeper understanding of the clinical manifestations of ES.

## Case report

### Clinical history

A 47-year-old female patient came to the outpatient clinic of FAHZU with a complaint of an asymptomatic right axillary mass for 3 months. Physical examination revealed a soft mass 4 cm in diameter in the right axilla without any other positive findings in the right breast. An axillary lymph node biopsy was conducted in a previous local hospital, and the histopathological results were suggestive of some metastatic carcinomas with an unknown primary lesion, while suspicion of lobular carcinoma of breast could not be ruled out.

**Fig. 1:**
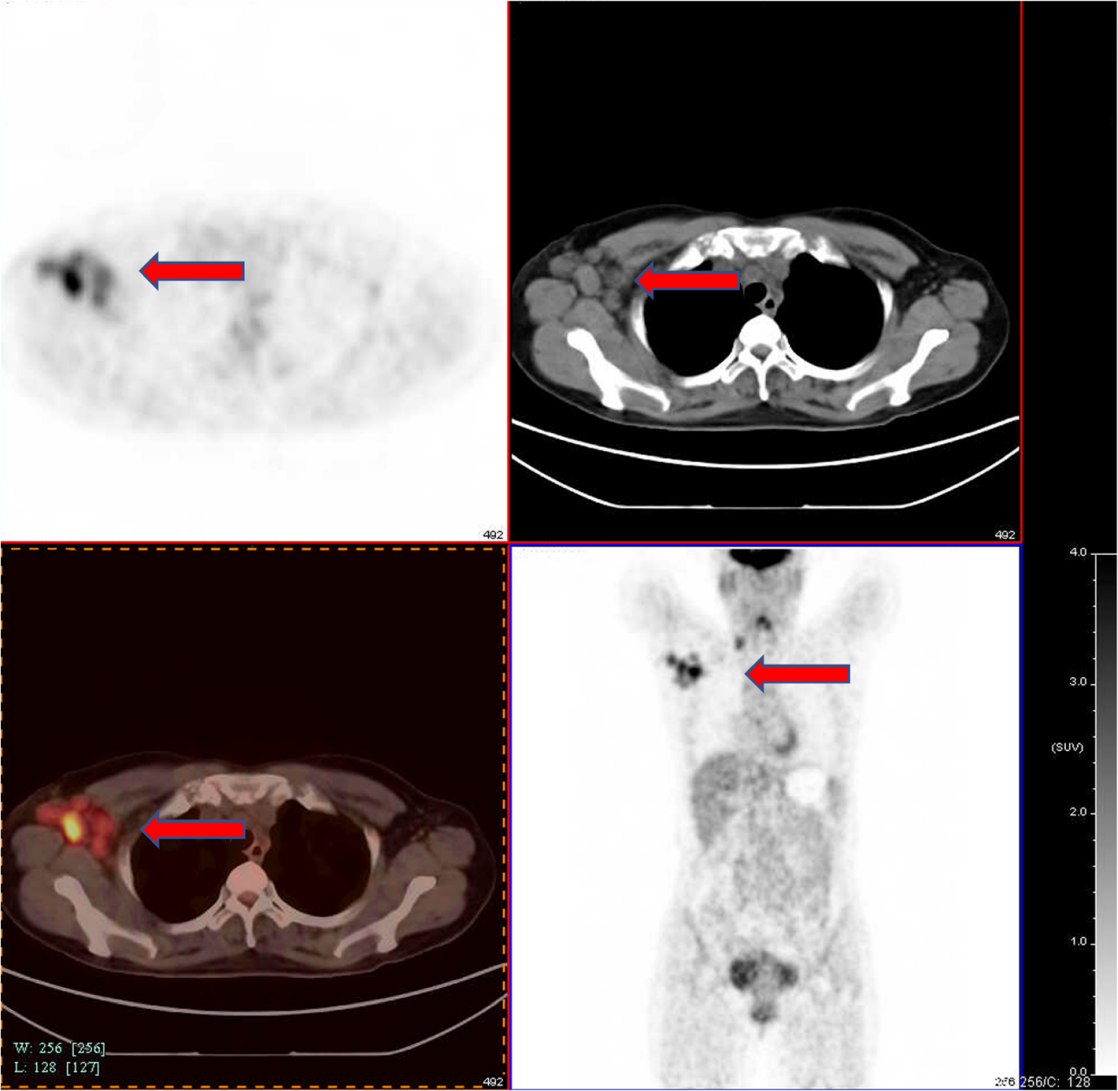
PET-CT before axillary surgery. The initial staging showed multiple lesions with significantly increased FDG intensity presenting in the right side axilla

In order to locate the primary lesion and other metastatic lesions, a PET-CT scan was performed in FAHZU, which reported multiple lesions with significantly increased FDG (18F-fluorodeoxyglucose) intensity presenting in the right side axilla. Meanwhile, the FDG (18F-fluorodeoxyglucose) was slightly elevated at the right elbow, considered as an inflammation or trauma (Fig. 1). Therefore, a surgical resection of the right axillary lymph nodes and right supraclavicular lymph nodes was conducted in FAHZU. The histopathology report showed that 31/32 lymph nodes in the right axilla and 10/10 lymph nodes in the right supraclavicula were positive for metastatic carcinoma. For the time being, suspicion of metastatic epithelial sarcoma and metastatic breast cancer could not be ruled out.

**Fig. 2:**
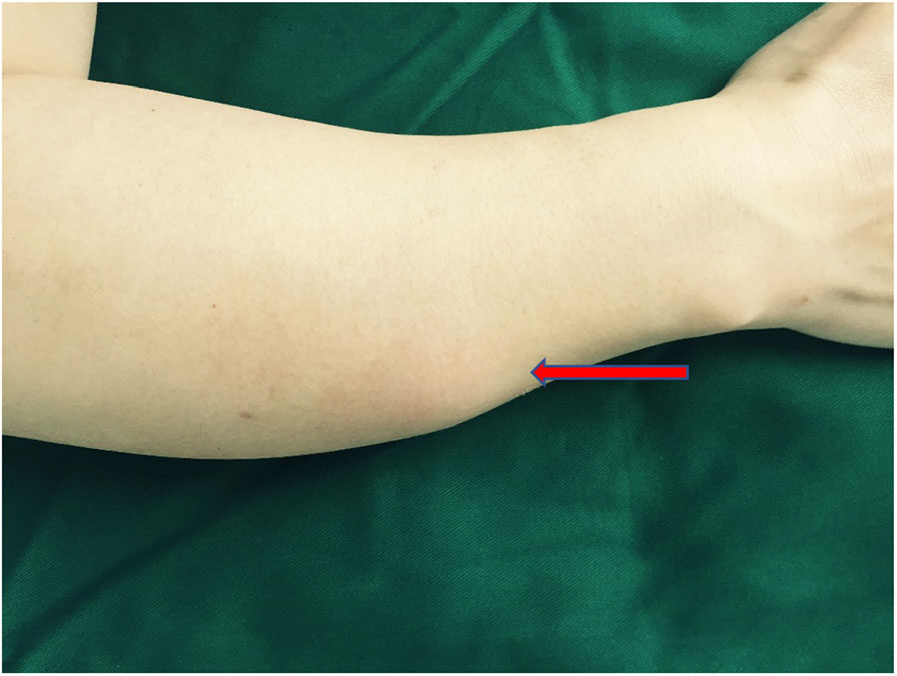
An asymptomatic mass on the right forearm

With more detailed medical history review and physical examination, a mass on the right forearm was found (Fig. 2). After a car accident 20 years ago, the mass had been growing slowly over the last decade without causing any pain or ulceration. There was also no abnormality of the right forearm X-ray in routine examination. An MRI scan was applied to the right forearm tumor and the report indicated that metastatic tumor could not be excluded. Therefore, a core needle biopsy (CNB) of the mass in the right forearm was conducted and the histopathology report suggested evidence of ES. So far, breast cancer was excluded and a surgery was performed to remove the right forearm tumor. After that, the patient received 6 cycles of chemotherapy with modified mesna, doxorubicin, ifosfamide and dacarbazine (MAID). Radiotherapy was denied to her after a multidisciplinary treatment (MDT).

**Fig. 3:**
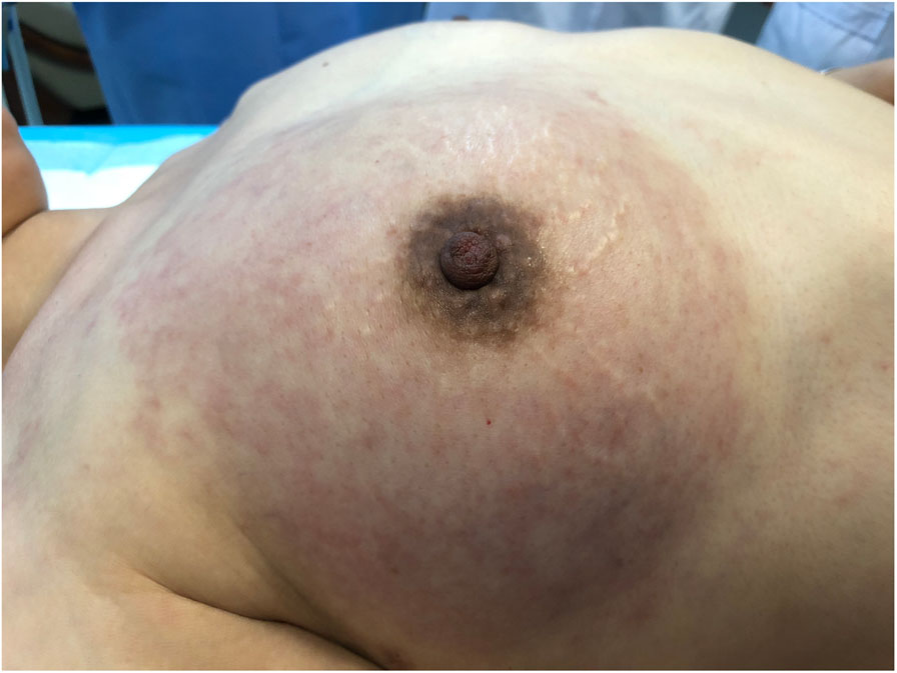
Ipsilateral Breast Metastasis. The right breast with whole redness looked like an inflammatory breast cancer ten months after the surgical resection of the right axillary lymph nodes and right supraclavicular lymph nodes

Ten months after operation, the patient came back with complaints of pain and redness in the right breast (Fig. 3). This time, there were still no positive findings in ultrasonography and PET-CT scan, while the blind CNB of the right breast showed whole breast metastasis of ES. In order to seek a useful therapeutic target, the patient was given a genetic testing. Regrettably, there was no positive result of the metastatic breast sample, sequenced by a 381-cancer-gene panel in a lab. She went through another 6 cycles of chemotherapy with Gemcitabine and Docetaxel, and took Apatinib for maintenance treatment. During the last follow-up (26 months after diagnosis), the disease was under control.

### Pathological findings

**Fig. 4:**
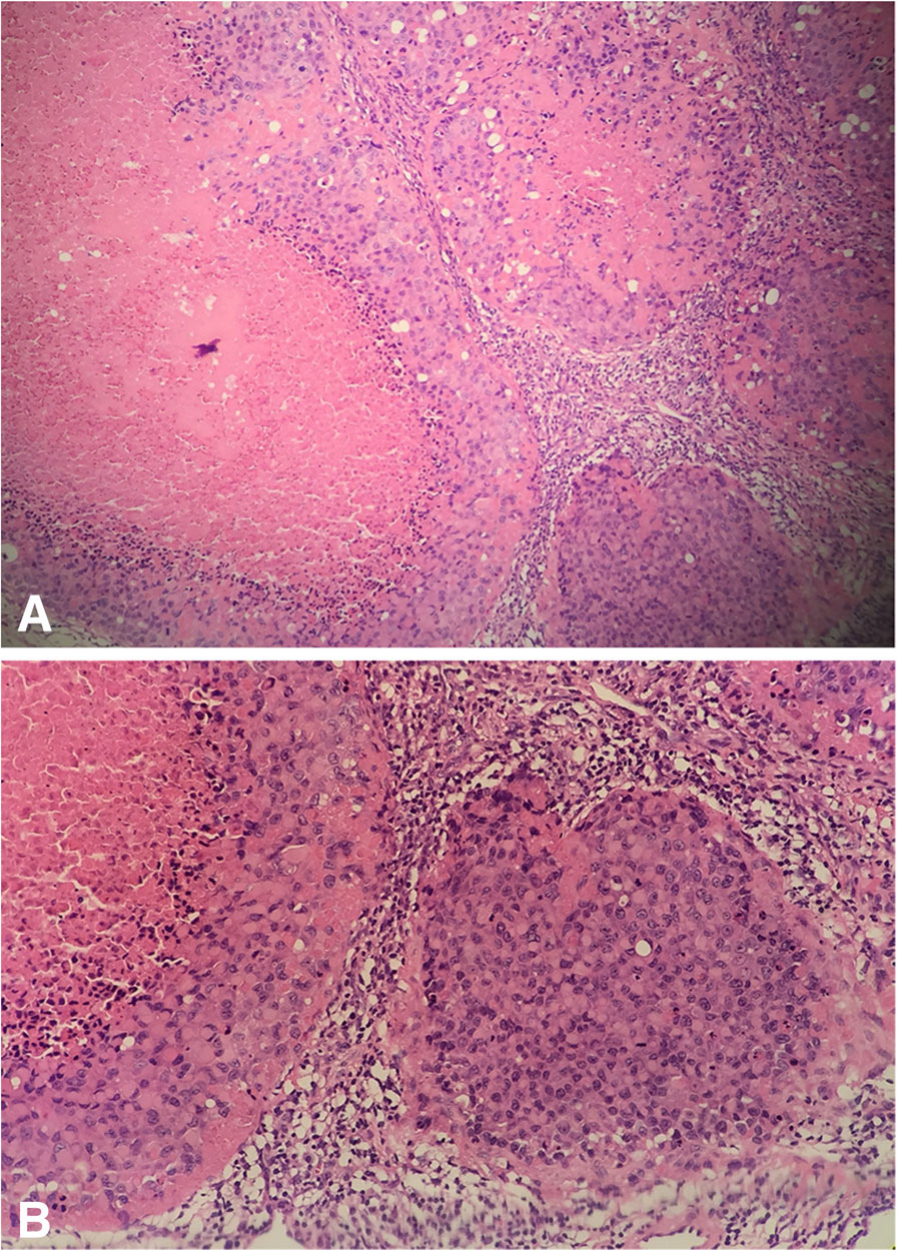
Microscopic findings of the right axillary lymph nodes. H&E staining indicated a poorly differentiated carcinoma (**a** × 100; **b** × 200)

In our case, pathology was the most important basis for excluding metastatic breast cancer. The axillary lymph nodes were poorly differentiated under microscope, which was why occult or metastatic breast cancer could not be ruled out in the first place. Histopathological examination showed that the neoplasm was identifiable by tumor nodules with central necrosis surrounded by medium-sized fairly uniform, plump epithelioid cells, and short spindle cells merging in the periphery with relatively abundant eosinophilic cytoplasm (Fig. 4). IHC showed CK (pan) (+), EMA (+), CK7 (−), CK8 (+), CK19(+), Vimentin (+), CD34 (++), CD31 (−), F8-R-Ag (−), CD68 (−), S-100 (−), MelanA (−), HMB45 (−), CK5/6 (−), P63 (−), ER (−), GATA-3 (−), E-adherin (−), P120 (+/−), TFE3 (−), MyoD1 (−), CD45 (LCA) (−).

**Fig. 5:**
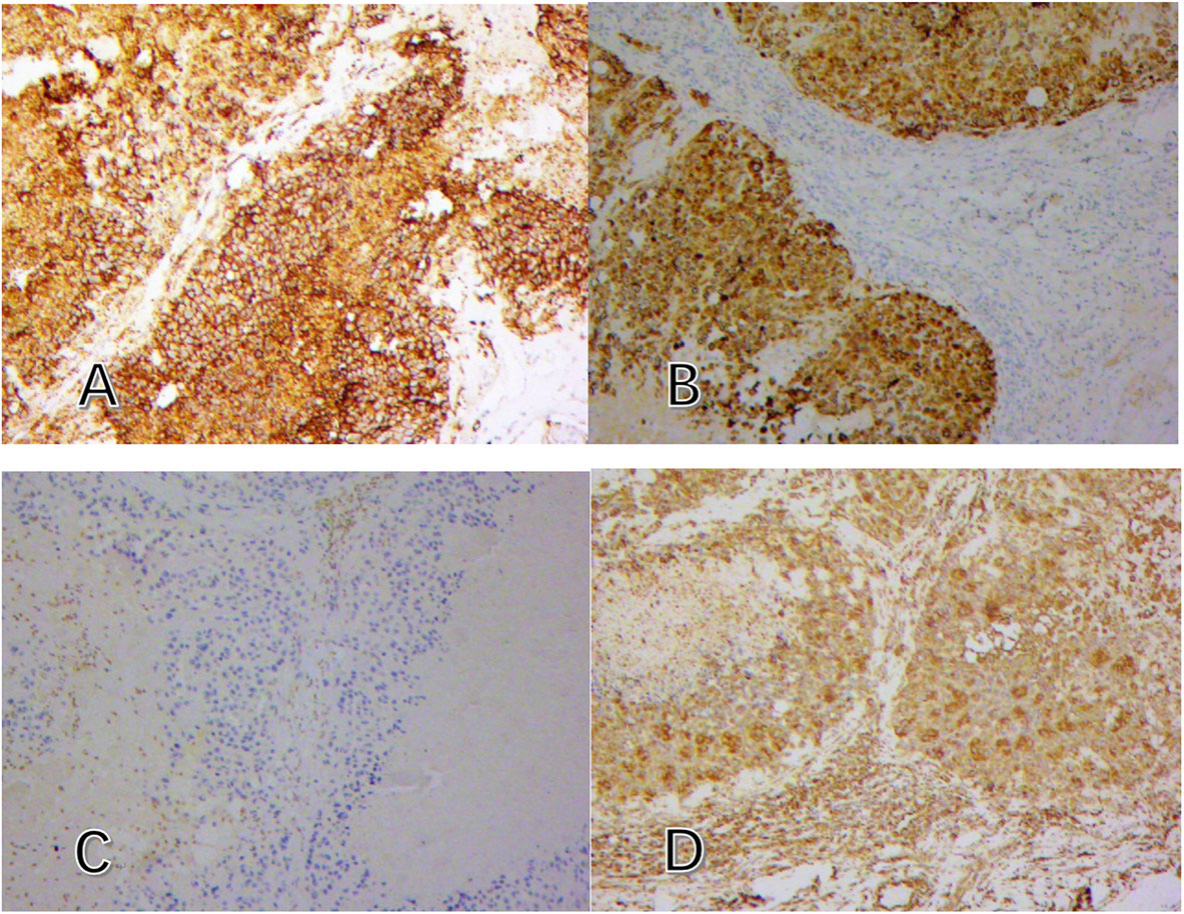
IHC of the right axillary lymph nodes. **a** CD34 positive (× 100). **b** CK (pan) positive (× 100). **c** INI1negative (× 100). **d** Vimentin positive (× 100)

**Fig. 6:**
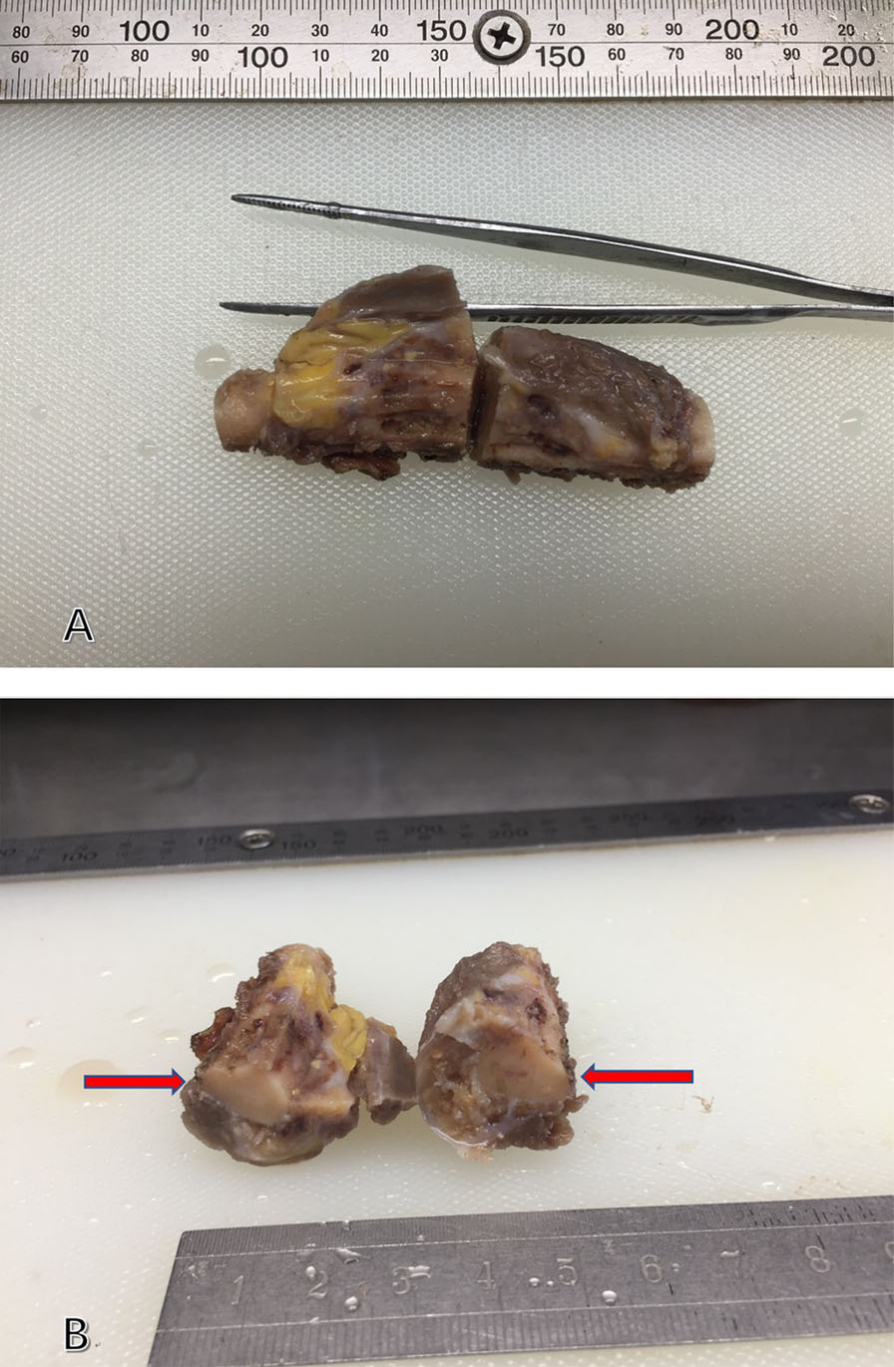
The resected specimen of the right forearm. **a** It was located beneath the dermis and could not be separated from the bone. **b** The size of the mass was 3.5 cm × 2 cm

After the right forearm mass was found, further IHC and Molecular Genetics of the lymph nodes were performed. The results suggested that all the neoplastic cells exhibited loss of INI1 protein and were negative for ERG gene rearrangement yet positive for EMA, CK8, CK19, Vimentin and CD34 (Fig. 5). Besides, the neoplastic cells were completely negative for high molecular weight cytokeratin CK5/6, P63. Proliferation index ki-67 was approximately 30%. After the forearm operation, visual inspection of the resected specimen showed that it was located beneath the dermis without cutaneous and ulcerous presentation and could not be separated from the bone (Fig. 6). Finally, we considered that the patient was with a conventional distal-type ES and the primary lesion was in the right forearm, excluding breast cancer.

## Discussion

Epithelioid sarcoma is a rare malignant soft tissue tumor with slow progression, which commonly occurs in distal extremities including fingers, hands and wrists. It can migrate to other organs such as lungs and form metastatic lesions through blood vessels and lymphatics [1]. Breast metastasis of ES has not been reported yet.

ES can be classified into two types: distal and proximal types. Distal-type ES usually shows characteristics of nodular aggregates, nests or cords of fairly uniform, plump epithelioid cells, with relatively abundant eosinophilic cytoplasm and distinct central zonal necrosis [4]. Cellular atypia is relatively mild in the primary lesion, compared with a greater degree of pleomorphism in recurrent or metastatic lesions. Proximal-type ES is usually featured by multinodular distributions and sheets of large polygonal cells with mildly to moderately pleomorphic vesicular nuclei and prominent nucleoli. Its key feature is Rhabdoid morphology, and the nodules often contain central necrosis. Although ES can occur in either proximal or distal sites, the tumors rarely contain all features of both types.

Characteristically, ES has immunoreactivity for vimentin and epithelial markers: low and high molecular weight cytokeratins, including keratin 8, keratin 19 and/or EMA. Half of the cases of ES are positive for CD34 [5] and vimentin. The staining pattern suggests that ES is a neoplasm of mesenchymal origin with epithelial dedifferentiation. Almost all cases of ES are negative for ERG gene rearrangement and according to detection by IHC or fluorescence in situ hybridization (FISH), 90% of malignant rhabdoid tumors show nuclear deficiency of INI1 [6, 7], with the inactivation of SMARCB1 [8].

ES needs to be distinguished from many other benign and malignant diseases. Benign lesions include granulomas [9], rheumatoid nodules, benign fibrous histiocytomas, and giant cell tumors of tendon sheath. Aside from the morphologic differences between benign lesions and ES, all benign lesions lack CK expression and most lack EMA, both of which are typically diffusely expressed in ES. Malignant diseases include tumors with epithelioid or rhabdoid morphology [5], such as malignant rhabdoid tumors (MRT), epithelioid malignant peripheral nerve sheath tumors (MPNST), myoepithelial tumors, primitive neuroectodermal tumors (PNET), extra-skeletal myxoid chondrosarcomas (EMC), epithelioid angiosarcomas, epithelioid hemangioendotheliomas (EHE), rhabdomyosarcomas, synovial sarcomas, malignant mesotheliomas, melanomas and so on.

In blood test of ES, Kato et al. documented that 10 of 11 (91%) patients had positive test results for CA-125 [10, 11]. In our case, results of all tumor markers were within normal range. New biomarkers are in need of discovery.

With regard to imaging, twice of PET-CT performed before and after operation were not sufficient to indicate all of the primary and metastatic lesions. Vien X et al. reported a case of proximal-type ES in scrotum with comprehensive staging and monitoring by PET-CT [12]. We speculated that PET-CT was not sensitive enough for the disease and other examinations like CT, MRI and CNB would assist the diagnosis. Moreover, the right axillary mass was considered as a metastasis from a malignant tumor of unknow origin and no mastectomy was performed at the first place. Unexpectedly, the patient developed ipsilateral breast metastasis ten months later which was similar to inflammatory breast cancer, without any other site involved. We wondered whether the metastasis would be avoided or delayed in case mastectomy was performed before. Pathologists showed us that the tumor of right breast puncture under the microscope was totally in the vessel. Since the main lymphatic pathway to the breast had been destroyed after axillary dissection, whole breast metastasis might be caused by residual focus in the breast, as called intra mammary metastasis, explaining the recurrence of the ipsilateral breast. We still wondered why there were no other metastases than the right breast.

As of treatments, Frezza AM et al. considered that anthracycline-based and gemcitabine-based treatments might be of choices [13]. The patient should not be denied of surgery to resect the breast if necessary. Localized radiotherapy should also be the treatment of choice for the disease. Although the genetic testing did not provide positive results, the levels of SWI/SNF complex [14] or PD-1/PD-L1 [15] in tissue could be evaluated, which may suggest genetic testing as a novel therapy for the disease. This case provides useful and informative clinical reference for breast doctors by presenting a case study of a rare malignant soft tissue tumor.

## Conclusion

In conclusion, epithelioid sarcoma is a rare malignant soft tissue tumor. Patients who come to the hospital reporting only enlarged axillary lymph node are easily misdiagnosed with occult breast carcinoma. Our case reported a woman with an axillary metastasis of ES in the right arm. She received a localized operation and classical chemotherapy, and an ipsilateral breast metastasis occurred ten months later. This rare but interesting case enables breast surgeons and pathologists to accumulate more experience of differential diagnosis of axillary mass for personalized treatment in clinical practice. Meanwhile, ipsilateral breast metastasis of ES we reported in our case encourages clinicians to attach greater importance to the tumor metastasis mechanism. For oncologists and surgeons, it is necessary to comprehensively assess the condition, think over the scope of surgery and actively treat the patients.

## Data Availability

All data generated or analyzed during this study are included in published article.

## Abbreviations

CK: Cytokeratin
CNB: Core needle biopsy
CT: Computer tomography
EHE: Epithelioid hemangioendothelioma
EMA: Epithelial membrane antigen
EMC: Extraskeletal myxoid chondrosarcomas
ES: Epithelioid sarcoma
FAHZU: First Affiliated Hospital of Zhejiang University
FDG: Fluorodeoxyglucose
FISH: Fluorescence in situ hybridization
GATA-3: GATA binding protein-3
H&E: Hematoxylin and eosin
HMB 45: Human melanoma black 45
IHC: Immunohistochemistry
INI 1: Integrase interactor 1
MAID: Modified mesna, doxorubicin, ifosfamide and dacarbazine
MDT: Multidisciplinary discussion
MPNST: Epithelioid malignant peripheral nerve sheath tumors
MRI: Magnetic resonance imaging
MRT: Malignant rhabdoid tumor
PET: Positron emission tomography
PNET: Primitive neuroectodermal tumor
TFE 3: Heavy-chain enhancer 3

## References

[1] Enzinger FM (1970). Epitheloid sarcoma. A sarcoma simulating a granuloma or a carcinoma. Cancer.

[2] Argenta PA, Thomas S, Chura JC (2007). Proximal-type epithelioid sarcoma vs. malignant rhabdoid tumor of the vulva: a case report, review of the literature, and an argument for consolidation. Gynecol Oncol.

[3] Guillou L, Wadden C, Coindre JM, Krausz T, Fletcher CD (1997). "proximal-type" epithelioid sarcoma, a distinctive aggressive neoplasm showing rhabdoid features. Clinicopathologic, immunohistochemical, and ultrastructural study of a series. Am J Surg Pathol.

[4] Rakheja D, Wilson KS, Meehan J, Schultz RA, Gomez AM (2005). "Proximal-type" and classic epithelioid sarcomas represent a clinicopathologic continuum: case report. Pediatr Dev Pathol.

[5] Fisher C (2006). Epithelioid sarcoma of Enzinger. Adv Anat Pathol.

[6] Kohashi K, Yamada Y, Hotokebuchi Y, Yamamoto H, Taguchi T, Iwamoto Y (2015). ERG and SALL4 expressions in SMARCB1/INI1-deficient tumors: a useful tool for distinguishing epithelioid sarcoma from malignant rhabdoid tumor. Hum Pathol.

[7] Martens JH (2011). Acute myeloid leukemia: a central role for the ETS factor ERG. Int J Biochem Cell Biol.

[8] Modena P, Lualdi E, Facchinetti F, Galli L, Teixeira MR, Pilotti S (2005). SMARCB1/INI1 tumor suppressor gene is frequently inactivated in epithelioid sarcomas. Cancer Res.

[9] Fanburg-Smith JC, Hengge M, Hengge UR, Smith JS, Miettinen M (1998). Extrarenal rhabdoid tumors of soft tissue: a clinicopathologic and immunohistochemical study of 18 cases. Ann Diagn Pathol.

[10] Kato H, Hatori M, Kokubun S, Watanabe M, Smith RA, Hotta T (2004). CA125 expression in epithelioid sarcoma. Jpn J Clin Oncol.

[11] Lee HI, Kang KH, Cho YM, Lee OJ, Ro JY (2006). Proximal-type epithelioid sarcoma with elevated serum CA 125: report of a case with CA 125 immunoreactivity. Arch Pathol Lab Med.

[12] Nguyen VX, Nguyen BD (2013). Proximal-type epithelioid sarcoma of the scrotum F-18-FDG PET/CT imaging. Clin Nucl Med.

[13] Frezza AM, Jones RL, Lo Vullo S, Asano N, Lucibello F, Ben-Ami E (2018). Anthracycline, gemcitabine, and Pazopanib in epithelioid sarcoma: a multi-institutional case series. JAMA Oncol.

[14] Jamshidi F, Bashashati A, Shumansky K, Dickson B, Gokgoz N, Wunder JS (2016). The genomic landscape of epithelioid sarcoma cell lines and tumours. J Pathol.

[15] Kim C, Kim EK, Jung H, Chon HJ, Han JW, Shin KH (2016). Prognostic implications of PD-L1 expression in patients with soft tissue sarcoma. BMC Cancer.

